# Spermatogonial stem cell regulation and spermatogenesis

**DOI:** 10.1098/rstb.2010.0026

**Published:** 2010-05-27

**Authors:** Bart T. Phillips, Kathrin Gassei, Kyle E. Orwig

**Affiliations:** Department of Obstetrics, Gynecology and Reproductive Sciences, Magee-Womens Research Institute, University of Pittsburgh School of Medicine, 204 Craft Avenue, Pittsburgh, PA, USA

**Keywords:** spermatogonial stem cells, spermatogenesis, fertility

## Abstract

This article will provide an updated review of spermatogonial stem cells and their role in maintaining the spermatogenic lineage. Experimental tools used to study spermatogonial stem cells (SSCs) will be described, along with research using these tools to enhance our understanding of stem cell biology and spermatogenesis. Increased knowledge about the biology of SSCs improves our capacity to manipulate these cells for practical application. The chapter concludes with a discussion of future directions for fundamental investigation and practical applications of SSCs.

## 1. Introduction

Spermatogonial stem cells (SSCs) are at the foundation of spermatogenesis and male fertility. Similar to other tissue-specific stem cells, SSCs are rare, representing only 0.03 per cent of all germ cells in rodent testes (Tegelenbosch & de Rooij 1993). This is because SSCs are heavily outnumbered by the differentiating spermatogonia, spermatocytes, spermatids and sperm that they produce (detailed below). SSCs are defined like all other stem cells, by their ability to balance self-renewing divisions and differentiating divisions. This balance maintains the stem cell pool and meets the proliferative demand of the testis to produce millions of sperm each day. Studies of SSCs are complicated because these cells are few in number and no unique identifying characteristics have been reported to date. We will review experimental tools used to study SSCs and summarize current knowledge about the characteristics and regulation of these adult tissue stem cells. We will focus primarily on rodent models, which have generated the majority of data about SSCs and the spermatogenic lineage.

## 2. Origin of the spermatogonial stem cell pool

SSCs arise from gonocytes in the postnatal testis, which arise from primordial germ cells (PGCs) during foetal development. PGCs are a transient cell population that is first observed as a small cluster of alkaline phosphatase-positive cells in the epiblast stage embryo at about 7–7.25 days post coitum (dpc). PGC specification is dependent on the expression of BMP4 and BMP8b from the extraembryonic ectoderm (Ginsburg *et al*. 1990; Lawson *et al*. 1999; Ying *et al*. 2001). During the formation of the allantois, the PGCs are passively swept out of the embryo before they start migrating via the hindgut to arrive at the indifferent gonad between 8.5 and 12.5 dpc in mice. PGCs replicate during the migratory phase and approximately 3000 PGCs colonize the genital ridges (Bendel-Stenzel *et al*. 1998). In the male gonad at about 13.5 dpc, PGCs give rise to gonocytes, which become enclosed in testicular cords formed by Sertoli precursor cells and peritubular myoid cells. Gonocyte is a general term that can be subcategorized into mitotic (M)-prospermatogonia, T1-prospermatogonia and T2-propsermatogonia (McCarrey 1993). M-prospermatogonia are located in the centre of the testicular cords, away from the basal membrane and continue proliferating until about 16.5 dpc of mouse development when they become T1-prospermatogonia and enter G0 mitotic arrest (McLaren 2003; Tohonen *et al*. 2003). Gonocytes resume proliferation during the first week after birth (marking their transition to T2-prospermatogonia), concomitant with migration to the seminiferous tubules basement membrane (Clermont & Perey 1957). T2-prospermatogonia that colonize the basement membrane give rise to the first round of spermatogenesis as well as establish the initial pool of SSCs that maintain spermatogenesis throughout postpubertal life (Kluin & de Rooij 1981; McCarrey 1993; Yoshida *et al*. 2006).

## 3. The spermatogenic cycle

**Figure 1.**
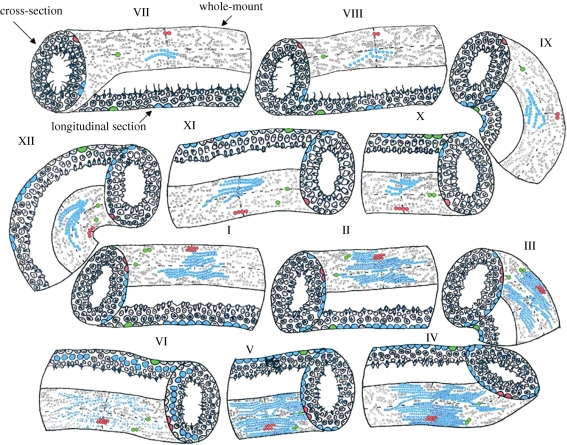
Mouse spermatogenic clone development by stage. The mouse spermatogenic cycle contains twelve stages (I–XII). Each stage is temporally unique, and the stages in the diagram represent the relative time each stage lasts in the mouse. Each stage in the diagram is shown in cross-sectional, longitudinal and whole-mount perspectives (labelled in stage VII). Three putative spermatogonial clones are highlighted in blue, red and green. The dotted lines in the whole-mount perspective indicate the planes of the cross section and longitudinal section views. For example, in stage VII, the red cell is in the vertical line and therefore appears in the cross-sectional view. A green cell is in the horizontal line, so is observed in the longitudinal section view. The development of three putative clones (blue, red and green) through one cycle of the seminiferous epithelium is shown. Stage VII: A_al-16_ (blue); A_pair_ (red); A_single_ (green); stage VIII: A1 (clone of 16) (blue); A_pair_ (red); A_single_ (green); stage IX: A2 (clone of 32) (blue); A_pair_ (red); A_single_ (green); stage X: A2 (clone of 32) (blue); A_al-4_ (red); A_pair_ (green); stage XI: A3 (clone of 64) (blue); A_al-4_ (red); A_single_ (x2) (green); stage XII: A3 (clone of 64) (blue); A_al-4_ (red); A_single_ (x2) (green); stage I: A4 (clone of 128) (blue); A_al-8_ (red); A_single_ and A_pair_ (green); stage II: intermediate spermatogonia (clone of 256) (blue); A_al-8_ (red); A_single_ and A_pair_ (green); stage III: intermediate spermatogonia (clone of 256) (blue); A_al-8_ (red); A_single_ and A_pair_ (green); stage IV: Type B Spermatagonia (clone of 512) (blue); A_al-8_ (red); A_single_ and A_pair_ (green); stage V: Type B Spermatagonia (clone of 512) (blue); A_al-8_ (red); A_single_ and A_pair_ (green); stage VI: primary spermatocytes (lifting off the basement membrane) (blue); A_al-8_ (red); A_single_ and A_pair (green)._

Spermatogenic lineage development is a complex process, but occurs in an orderly manner, referred to as the spermatogenic cycle (Clermont 1972), which is divided in a species-specific number of stages or cell associations (i.e. 12 stages in the mouse (Oakberg 1956*a*,*b*) and 14 stages in the rat (Leblond & Clermont 1952*a*,*b*)). This synchronized spermatogenic development may be facilitated by incomplete cytokinesis during mitotic divisions that lead to maintenance of cytoplasmic bridges among germ cells. Proteins and messenger RNAs are exchanged via the cytoplasmic bridges and may help in coordinating the synchronized development of germ cell clones (Braun *et al*. 1989). Each stage is characterized by a combination of the types of spermatogonia, spermatocytes and spermatids that synchronously proceed through the spermatogenic process (figure 1). For example, the basement membrane of a stage V seminiferous tubule depicted in figure 1 is mostly filled with preleptotene primary spermatocytes (blue cells of a large clone). By stage VI, these spermatocytes migrate off the basement membrane and will be replaced by spermatogonia. Thus, stage V can be distinguished from stage VI by the presence or absence of spermatocytes on the basement membrane. The duration of each stage is precisely timed, and the complete spermatogenic cycle was determined to be around 8.6 days in the mouse (Oakberg 1956*b*), and 12.8 days in the rat (Hilscher *et al*. 1969). One complete cycle (12 stages) of the mouse seminiferous epithelium is depicted in figure 1.

## 4. Spermatogenic lineage development

In order to understand the regulation of spermatogonial stem cells, it is important to understand them in the context of the spermatogenic lineage that they produce. Spermatogonia are primitive diploid germ cells, located on the basement membrane of the seminiferous tubules. Three types of spermatogonia were initially described based on their nuclear morphology (Roosen-Runge & Giesel 1950; Clermont & Leblond 1953; Monesi 1962). Type A spermatogonia were considered the most primitive because heterochromatin is absent from the nucleus, a general characteristic of undifferentiated cells. The nuclei of intermediate type spermatogonia contain a small amount of heterochromatin and type B spermatogonia contain a large amount of heterochromatin, indicating a more differentiated state.

Histological staining of whole-mount preparations of seminiferous tubules provided additional level of detail about spermatogonial morphometry compared with tissue sections alone and broadened the knowledge of the spermatogonial cell types in the testis. To facilitate the following discussion, figure 1 depicts one compete cycle of the mouse seminiferous epithelium and represents whole-mount perspective as well as corresponding cross-section and longitudinal section perspectives. Figure 1 traces the development of three putative clones (green, red and blue) through one cycle of the seminiferous epithelium. Based on whole-mount examination of seminiferous tubules, Huckins & Oakberg (Huckins 1971*c*; Oakberg 1971) reported that undifferentiated type A spermatogonia can be subdivided into A_single_ (A_s_), A_paired_ (A_pr_) and A_aligned_ (A_al_) spermatogonia, which differ only in their topographical arrangement on the seminiferous tubule basement membrane. When an A_s_ (see green clone in figure 1, stage VII) spermatogonium divides, it produces an A_pr_ that either (i) completes cytokinesis to produce two new A_s_ spermatogonia (self-renewing division, see green clone in figure 1, stages IX, X and XI) or (ii) remains connected by an intercellular cytoplasmic bridge and produces a chain of four A_al_ spermatogonia at the next division (differentiating division, see red clone in figure 1, stages IX and X). Further cell divisions lead to the formation of chains of 8, 16 and sometimes 32 A_al_ spermatogonia (see red clone in stages XII and I and blue clone in stage VII, figure 1). Chains of 4–16 A_al_ are generally considered committed to the differentiation process. Thus, the stem cell pool includes A_s_ and at least some A_pr_ spermatogonia. Some have argued that stem cell potential may extend to larger clones (e.g. A_al_4 or beyond; (Yoshida *et al*. 2007*a*; Morimoto *et al*. 2009)), but this is difficult to confirm experimentally. Note that while each clone can be observed in histological sections as well as in whole-mount preparations of seminiferous tubules, clone size can only be observed in the whole-mount preparations (figure 1).

A_s_, A_pr_ and smaller chains of four A_al_ spermatogonia are evenly distributed along the seminiferous epithelium (Huckins 1971*a*,*b*; Tegelenbosch & de Rooij 1993). Larger chains of A_al_ (8, 16 and 32) become differentiating A1 spermatogonia between stages IV and VIII of the seminiferous epithelium (there is no cell division at this transition, see blue clone in figure 1, stages VII and VIII) and these give rise to A2 spermatogonia at stage IX (see blue clone in figure 1, stage IX). Thus, in contrast to undifferentiated spermatogonia, differentiating spermatogonia (A1, A2, A3, A4, intermediate and B) divide in a synchronized manner and are found at specific stages of the seminiferous epithelium (for detailed description see Oakberg 1971). B spermatogonia give rise to primary spermatocytes that progress into meiosis. Two meiotic divisions lead to the formation of secondary spermatocytes and haploid spermatids respectively, which undergo 16 steps of morphological changes to finally become spermatozoa ready to be released from the seminiferous epithelium (Oakberg 1956*a*).

An alternative to the A_s_ model of SSC self-renewal described above is the A0/A1 model (Clermont & Bustos-Obregon 1968; Dym & Clermont 1970; Clermont & Hermo 1975). This model is very similar to the A_dark_ and A_pale_ model that has been used to describe stem cell activity in non-human primates (Clermont & Leblond 1959; Clermont & Antar 1973). Briefly, A0 spermatogonia were observed as singles or pairs of cells that were present at all stage of the seminiferous epithelium. Mitotic figures were rarely observed in these cells, so they were considered ‘reserve stem cells’ not contributing to steady-state spermatogenesis. These reserve stem cells are only activated when spermatogenesis is destroyed by toxic insult (i.e. radiation). The ‘active stem cell’ pool is comprised of A1–A4 spermatogonia. When A4 spermatogonia divide, they give rise either to new A1 spermatogonia (self-renewal) or to intermediate spermatogonia (differentiation). While there continues to be vigorous debate about the merits of the A_s_ versus the A0/A1 models, the A_s_ model is currently favoured by most investigators in the field and will be the basis for further discussion of spermatogonial self-renewal in this review.

## 5. Experimental tools for studying spermatogonial stem cells

As discussed above, experimental investigation of SSCs is complicated because these cells are rare and are difficult to distinguish from the differentiating progeny that they produce. Whole-mount analyses of seminiferous tubules help in distinguishing A_s_ from A_pr_ and A_al_ spermatogonia, but there is continuing debate about whether the stem cell pool is restricted to A_s_ or might be expanded to include A_pr_ and some A_al_ (Nakagawa *et al*. 2007; Yoshida *et al*. 2007*a*). Thus, the only way to definitively identify an SSC is by observing its biological capacity to produce and maintain spermatogenesis in a transplant paradigm.

## 6. Spermatogonial stem cell transplantation

**Figure 2.**
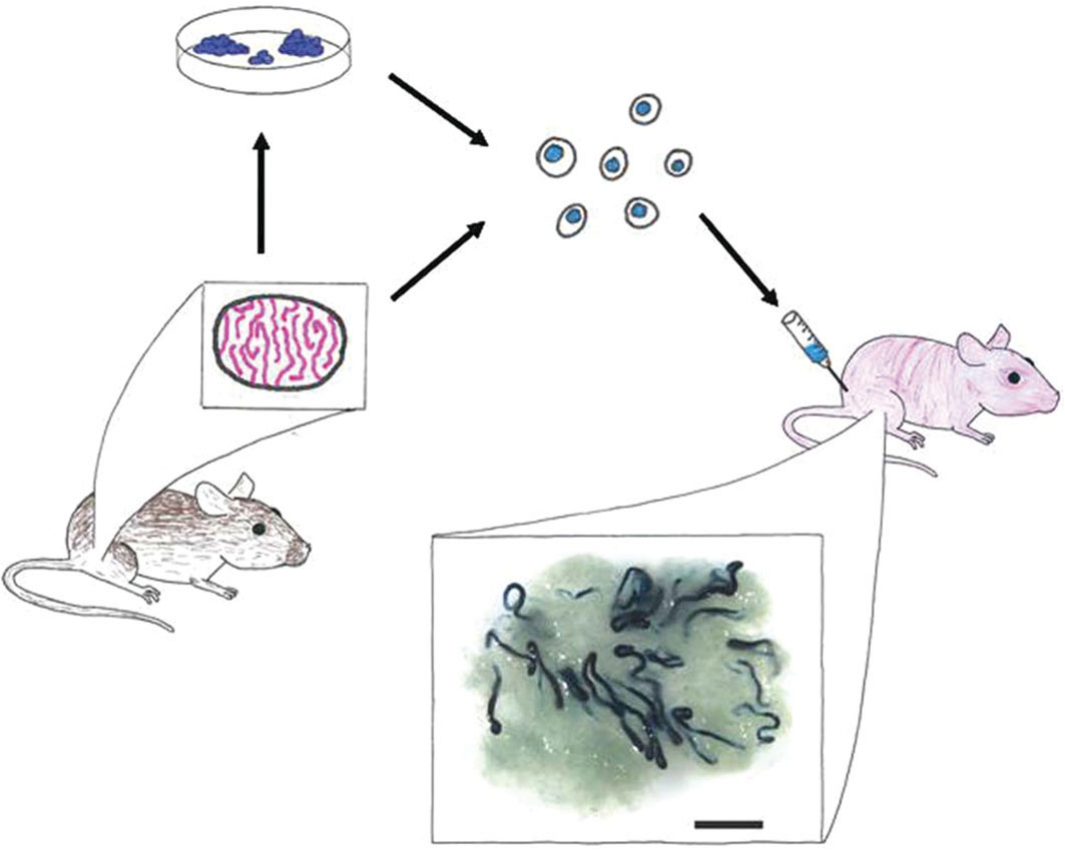
Spermatogonial stem cell (SSC) transplant assay. The functional analysis of SSCs is a retrospective assay of spermatogenic function. In this example, cells are isolated from a *lac*Z donor mouse testis and digested to produce a single cell suspension. Cells can then be maintained in culture or injected into the testes of an infertile recipient mouse. Recipient testes are typically analysed two to three months after transplantation for donor spermatogenesis (blue colonies in this example). A typical recipient testis is shown with blue colonies of donor-derived spermatogenesis (scale bar, 2 mm).

A technique for transplanting SSCs was first described by Brinster and colleagues in 1994 (Brinster & Avarbock 1994; Brinster & Zimmermann 1994). Briefly, germ cells are isolated from the testes of donor animals and transplanted into the testicular seminiferous tubules of infertile recipients, where they produce normal colonies of spermatogenesis and functional sperm (figure 2). Infertility of recipients is because of genetic mutation (i.e. *W* mutant mice, (Ogawa *et al*. 2000)) or induced experimentally (e.g. busulphan treatment (Brinster & Zimmermann 1994)). In mice, these studies are facilitated by the availability of transgenic donors (e.g. *lac*Z and *GFP*) with germ cells that can be readily identified in the testes of non-transgenic recipients. By definition, only a stem cell can produce and maintain a colony of spermatogenesis and each colony arises from the clonogenic proliferation and differentiation of a single SSC (Dobrinski *et al*. 1999; Zhang *et al*. 2003; Kanatsu-Shinohara *et al*. 2006). Therefore, the SSC transplantation technique provides a quantitative functional assay to characterize stem cell activity in any donor cell population. SSC transplantation remains the gold standard method for identifying SSCs, but this approach can be technically challenging. In addition, SSC transplantation is a retrospective assay with an inherent two to three months timeframe between transplant and analysis. To accelerate investigations of SSCs, Nagano and co-workers recently suggested that the SSC culture system (described below) may provide a shorter term, *in vitro* assay for SSCs (Yeh *et al*. 2007). However, culture does not assess regenerative activity.

## 7. Dissecting the molecular phenotype of spermatogonial stem cells

Fluorescence-activated cell sorting (FACS), combined with SSC transplantation is a powerful tool that has enabled investigators to systematically characterize cell surface molecules of SSCs. This experimental approach is patterned after similar studies to characterize and enrich haematopoietic stem cells (Spangrude 1989; Smith *et al*. 1991; Osawa *et al*. 1996). Briefly, a heterogeneous testis cell suspension is stained with a fluorescent-conjugated antibody that recognizes a cell surface antigen. Marker^+^ and marker^−^ cells are fractionated by FACS and each fraction is transplanted into the seminiferous tubules of infertile recipient mice to determine the relative stem cell activity. The first application of this approach for characterizing SSCs was reported by Shinohara and co-workers, who demonstrated that SSCs specifically bind to laminin-coated plates. The laminin-binding cells were enriched for β1-integrin, making this surface molecule a candidate for enriching SSCs (Shinohara *et al*. 1999). Subsequent transplantation of magnetic-activated cell-sorted (MACS) and FACS-sorted testis fractions indicated that SSCs express β1-integrin and α6-integrin, but are negative for αv-integrin and the c-KIT receptor tyrosine kinase (Shinohara *et al*. 1999, 2000). Based on several similar studies, mouse SSCs can now be described by the cell surface phenotype, α6-Integrin (CD49f)^+^, β1-Integrin (CD29)^+^, THY-1 (CD90)^+^, CD9^+^, GFRα1^+^, CDH1^+^, αv-Integrin (CD51)^−^, c-KIT (CD117)^−^, major histocompatibility complex class I (MHC-I)^−^, CD45^−^ (Shinohara *et al*. 1999, 2000; Kubota *et al*. 2003; Kanatsu-Shinohara *et al*. 2004*b*; Buageaw *et al*. 2005; Fujita *et al*. 2005; Hofmann *et al*. 2005*b*; Lo *et al*. 2005; Tokuda *et al*. 2007, table 1). Using combinations of positive and negative markers, it is now possible to achieve significant enrichment (100- to 200-fold) of mouse SSCs (Shinohara *et al*. 2000; Kubota *et al*. 2003). However, it should be noted that none of these markers are exclusive to SSCs and no marker or combination of markers has produced a pure population of SSCs. Also, while FACS and MACS sorting followed by transplantation are powerful tools for characterizing the cell surface phenotype of SSCs, this approach has limited utility for characterizing cytoplasmic or nuclear markers.

**Table 1.** Germ cell markers in the rodent testis.

			germ cell type									
germ cell markers in the rodent testis	experimental evidence	transplantable SSC?^a^	As	Apr	Aal	A1–4	In	B	Spc	RS	ES	references
c-kit	Mu, RT–PCR, ISH, IHC, Tr	no			X	X	X	X	X	X		Manova *et al*. (1990), Yoshinaga *et al*. (1991), Schrans-Stassen *et al*. (1999) and Shinohara *et al*. (2000)
GCNA1	WB, IHC	not tested	X	X	X	X	X	X	X	X		Enders & May (1994)
VASA (MvH)	ISH, WB, IHC, KO	not tested	X	X	X	X	X	X	X	X		Fujiwara *et al*. (1994), Tanaka *et al*. (2000) and Toyooka *et al*. (2000)
EE2 antigen	WB, IHC,	not tested	X	X	X	X	X	X	X			Koshimizu *et al*. (1995)
DAZL	RT–PCR, NB, ISH, KO, IHC	not tested	X	X	X	X	X	X	X			Cooke *et al*. (1996), Niederberger *et al*. (1997) and Ruggiu *et al*. (1997)
Stra8	RT–PCR, ISH, IHC, WM, TG, MACS, Tr	yes	X	X	X	X	X	X	X	X	X	Oulad-Abdelghani *et al*. (1996), Giuili *et al*. (2002) and Antonangeli *et al*. (2009)
*α*6-integrin (CD49f)	FC, MACS, Tr	yes	X	X	X	X	X	X	X			Shinohara *et al*. (1999, 2000)
*β*1-integrin (CD29)	FC, MACS, Tr	yes	X	X	X	X	X	X				Shinohara *et al*. (1999) and Kanatsu-Shinohara *et al*. (2008)
Epcam	IHC, MACS	not tested	X	X	X	X	X	X				Anderson *et al*. (1999), van der Wee *et al*. (2001) and Tokuda *et al*. (2007)
Pou5f1 (Oct4)	IHC, WM, TG, FC, Tr, ISH	yes	X	X	X						X	Pesce *et al*. (1998), Yoshimizu *et al*. (1999), Ohbo *et al*. (2003) and Ohmura *et al*. (2004)
GFR-*α*1	TG, ISH, IHC, MACS, TR, WM	yes	X	X	X							Meng *et al*. (2000), Dettin *et al*. (2003), Buageaw *et al*. (2005) and Grisanti *et al*. (2009)
CD24	FC	not tested	X	X	X							Kubota *et al*. (2003)
Thy1 (CD90)	FC, TR	yes	X	X	X							Kubota *et al*. (2003)
Nanos2	ISH, KO, WB, RT–PCR, Tg, IHC, TR, WM	yes	X	X								Tsuda *et al*. (2003), Suzuki *et al*. (2007, 2009) and Sada *et al*. (2009)
Nanos3	NB, ISH, KO, WB, RT–PCR, Tg, IHC, WM	not tested	X	X	X							Tsuda *et al*. (2003), Suzuki *et al*. (2007, 2009) and Lolicato *et al*. (2008)
CD9	FC, IHC, MACS, Tr	yes	X	X	X	X	X	X				Kanatsu-Shinohara *et al*. (2004*b*)
EGR3	IVC, IHC	not tested	X	X								Hamra *et al*. (2004)
Ngn3	ISH, TG, WM, IHC, Tr	yes (approx. 10% of SSCs)	X	X	X				X			Yoshida *et al*. (2004, 2006) and Raverot *et al*. (2005)
PLZF	Mu, KO, Tr, WM, FC, ISH, IHC	yes	X	X	X							Buaas *et al*. (2004), Costoya *et al*. (2004) and Grisanti *et al*. (2009)
RBM	RT–PCR, IHC	not tested	X	X	X	X						Jarvis *et al*. (2005)
Sox-3	KO, IHC	not tested	X	X	X							Raverot *et al*. (2005)
TAF4B	KO, IHC	not tested	X	X	X	X	X	X	X		X	Falender *et al*. (2005)
Bcl6b	siRNA	not tested	X	X	X							Oatley *et al*. (2006)
Numb	NB, WB, IHC	not tested	X	X	X	X	X	X	X			Corallini *et al*. (2006)
Lrp4	WB, IHC	not tested	X	X	X	X	X	X	X	X	X	Yamaguchi *et al*. (2006)
Ret	IHC, MACS, Tr	no	X	X	X							Ebata *et al*. (2005) and Naughton *et al*. (2006)
Sohlh1	KO, RT–PCR, IHC,	not tested			X	X	X	X	X			Ballow *et al*. (2006*a*)
Sohlh2	RT–PCR, IHC	not tested	X	X	X							Ballow *et al*. (2006*b*)
CDH1 (CD324)	IHC, WM, MACS, Tr	yes	X	X	X							Tokuda *et al*. (2007)
GPR125	TG, FC, IVC, Tr	yes	X	X	X							Seandel *et al*. (2007)
Nucleostemin	TG, IHC, FC, Tr, IVC, siRNA	yes	X	X	X	X	X	X	X			Ohmura *et al*. (2008)
UTF1	RT–PCR, IHC	not tested	X	X	X							van Bragt *et al*. (2008)
Lin28 (Tex17)	IHC, WM, WB, siRNA	not tested	X	X	X							Zheng *et al*. (2009)

As, A single spermatogonia; Apr, A paired spermatogonia; Aal, A aligned spermatogonia; A1–4, differentiating type A1 to A4 spermatogonia; In, intermediate type spermatogonia; B, type B spermatogonia; Spc, spermatocytes; RS, round spermatids; ES, elongated spermatids; Mu, mutant mouse; TG, transgenic mouse; KO, Knockout mouse; Tr, germ cell transplantation; IHC, immunohistochemistry; WM, whole mount immunostaining; FC, flow cytometry (including FACS); IVC, *in vitro* culture; WB, Western blot; NB, Northern blot; ISH, *in situ* hybridization; RT–PCR, reverse transcriptase–PCR; siRNA, *in vitro* knockdown experiment using siRNA; MACS, magnetic-activated cell sorting.

^a^As determined by the spermatogonial stem cell transplantation assay.

Genetic mouse models in which GFP expression is driven by a promoter from a putative SSC gene provide an alternative approach for characterizing SSCs. For example, Schöler and co-workers reported that the OCT-4 transcription factor is expressed by gonocytes and type A spermatogonia of newborn, pup and adult mouse testes (Pesce *et al*. 1998). This group subsequently characterized an 18 kb promoter/enhancer fragment of the *Oct-4* gene that directed faithful expression of *lac*Z and *GFP* transgenes (Yeom *et al*. 1996; Yoshimizu *et al*. 1999). The Oct-4–GFP mouse is a valuable tool that enabled FACS-based isolation and transplantation of *Oct4* expressing germ cells from a heterogeneous testis cell suspension (Ohbo *et al*. 2003; Ohmura *et al*. 2004). Stem cell activity was significantly enriched in the *Oct4* expressing (GFP+) population compared with the *Oct4* negative (GFP−) population of mouse testis cells (Ohmura *et al*. 2004). Interestingly, gonocytes and pre-spermatogonia from neonatal mice with an Oct4–EGFP^+^/c-Kit^−^ phenotype had a greater repopulation capacity than Oct4–EGFP^+^/c-Kit^+^ cell fractions (Ohbo *et al*. 2003). These data suggest that there is molecular heterogeneity among pre-spermatogonia. This observation is consistent with reports suggesting that some gonocytes/pre-spermatogonia establish the initial pool of SSCs, while other gonocytes/pre-spermatogonia differentiate to produce the first round of spermatogenesis (Kluin & de Rooij 1981; Yoshida *et al*. 2006).

Transgenic and conditional knock-in approaches were recently used to demonstrate that neurogenin 3 (*Ngn3*) is expressed by the earliest spermatogonia (Yoshida *et al*. 2004), including at least 11 per cent of transplantable SSCs (Nakagawa *et al*. 2007). The fact that *Ngn3* was not expressed by all transplantable stem cells in that study provides additional evidence that there may be heterogeneity among SSCs. A conditional knock-in approach was also used to demonstrate that *Nanos2* is expressed by SSCs (Sada *et al*. 2009). Finally, transgenic models suggest that *Stra-8* (stimulated by retinoic acid-8) is expressed by undifferentiated spermatogonia, including SSCs (Giuili *et al*. 2002; Guan *et al*. 2006; Sadate-Ngatchou *et al*. 2008), although the transplant data in the *Stra-8* studies were limited.

Knock-out models have also been used to demonstrate that specific genes/proteins are required for SSC function. Male mice carrying the *luxoid* (*lu*) mutation are subfertile and show abnormal sperm development. Progression of infertility is caused by gradual loss of SSCs (Buaas *et al*. 2004). The mutation was shown to affect the *Zfp145* locus, which encodes the transcriptional repressor PLZF (promyelocytic leukaemia zinc-finger). PLZF is expressed during embryogenesis and plays a crucial role during limb and axial skeletal patterning. Targeted disruption of *Zfp145* resulted in a testicular phenotype similar to that of luxoid mutant mice (Costoya *et al*. 2004). In the testis, PLZF expression is restricted to A_s_, A_pr_ and A_al_ undifferentiated spermatogonia, including SSCs as demonstrated by transplantation experiments of testicular cells from *luxoid* or PLZF^−/−^mice that failed to initiate donor-derived spermatogenesis in recipient mice (Buaas *et al*. 2004; Costoya *et al*. 2004). A possible role for PLZF in spermatogonia could be the maintenance of an undifferentiated state (Filipponi *et al.* 2007), similar to the role suggested for Plzf in haematopoietic precursor cells (Reid *et al*. 1995).

Similar knock-out and over-expression studies implicate glial cell line-derived neurotrophic factor (GDNF) and its receptor GFRα1 in stem cell self-renewal (Meng *et al*. 2000). GDNF signalling has since been shown to be required for *in vitro* expansion of SSCs, and it has been demonstrated that a combination of GDNF and soluble GFRα1 is most favourable for the self-renewal of SSCs *in vitro* (Kubota *et al*. 2004*a*,*b*; see below). Finally, knock-out studies implicate *Sox3* in the differentiation of the earliest germ cells (Raverot *et al*. 2005). The latter study indicated that *Ngn3* expression is dependent on SOX3 and suggested that SOX3 may act directly or indirectly through *Ngn3* to regulate spermatogonial differentiation (Raverot *et al*. 2005).

**Figure 3.**
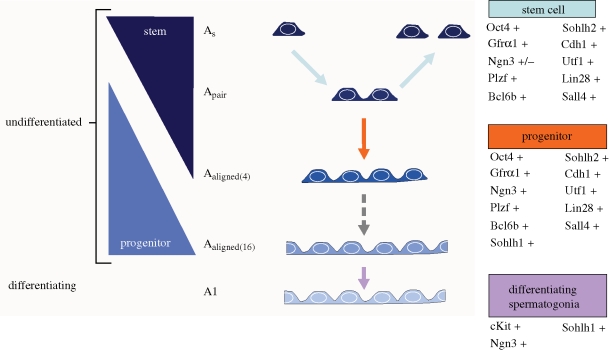
Genes expressed by stem, progenitor and differentiating spermatogonia. The A_s_, seen at the top of the diagram, is responsible for self renewal and differentiation. Self-renewal is represented here by the A_pair_ dividing to form two A_s_. Differentiation is indicated by colour change (from dark to light) and the lengthening chain of germ cells. Genes are listed with their expression at the given stages of spermatogonial development. While stem cell activity is considered to reside in the pool of A_s_ spermatogonia, the tapered triangle on the left indicates that stem cell activity may extend to A_pr_ and some A_al_ spermatogonia.

**Figure 4.**
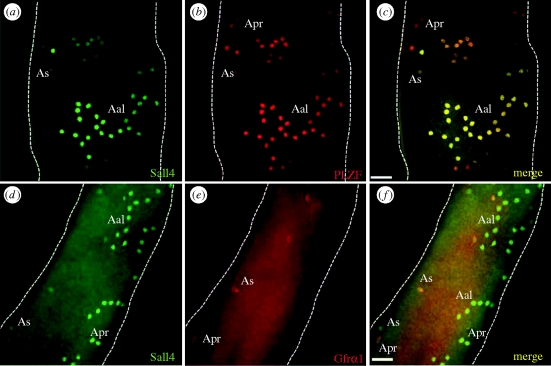
Immunofluorescent co-staining of adult mouse whole-mount seminiferous tubules. (*a*) SALL4 labels undifferentiated A_s_, A_pr_ and A_al_ spermatogonia. (*b*) PLZF labels undifferentiated A_s_, A_pr_ and A_al_ spermatogonia. (*c*) Merged picture from (*a*,*b*). SALL4 and PLZF are mostly co-expressed in undifferentiated spermatogonia. (*d*–*f*) Co-staining of SALL4 and GFRα1 reveals heterogeneity within the population of undifferentiated spermatogonia. GFRα1 expression appears more restricted than SALL4 or PLZF. Scale bar, 50 µm.

In addition to data derived from flow cytometry, genetic models and transplantation, immunohistochemistry in tissue sections or intact seminiferous tubules (whole mount) has been widely used to investigate the expression patterns of various proteins in the male germ lineage. In this context, a candidate SSC marker would be expressed by cells located on the basement membrane of seminiferous tubules and be co-expressed with confirmed markers of SSCs. This histochemical approach is most convincing in whole-mount preparations of seminiferous tubules in which it is possible to correlate marker expression with clone size (i.e. A_s_, A_pr_, A_al_). Several established markers of stem, progenitor and differentiating spermatogonia are listed in figure 3. Here we define progenitors as undifferentiated spermatogonia that are committed to differentiate. An example of this approach is shown in figure 4 for the putative SSC marker, Spalt-like 4 (SALL4). SALL4 is a zinc finger transcription factor that is expressed in the inner cell mass of the late blastocyst in a pattern similar to OCT4 and SOX2 (Elling *et al*. 2006). *In vitro*, SALL4 stimulates embryonic stem (ES) cell proliferation (Sakaki-Yumoto *et al*. 2006) and maintains pluripotency by repressing trophectoderm differentiation (Yuri *et al*. 2009), possibly by binding the *Oct-4* proximal promoter (Zhang *et al*. 2006) and by interacting with NANOG (Wu *et al*. 2006). Thus, SALL4 is an important stemness factor and together with OCT-4, SOX2 and NANOG constitutes a tightly regulated transcription circuit important for stem cell pluripotency (Lim *et al*. 2008; Yang *et al*. 2008). Postnatally, *Sall4* expression is restricted to the gonads and is expressed by isolated spermatogonia (Wang *et al*. 2001; *Sall4* was identified as testis-expressed gene 20 (*Tex20*) in that paper). Co-stained whole-mount seminiferous tubules (figure 4) indicated that SALL4 is expressed by single, paired and aligned cells on the seminiferous tubule basement membrane and overlaps with consensus SSC markers, PLZF (figure 4*a*–*c*) and GFRα1 (figure 4*d*–*f*). However, these whole-mount immunohistochemistry results highlight the heterogeneity among undifferentiated spermatogonia, including A_s_ spermatogonia (see figure 4*f* with examples of SALL4^+^/GFRα1^−^ and SALL4^+^/GFRα1^+^ A_s_ spermatogonia). GFRα1 appears to have the most restricted expression (limited to singles, pairs and chains of four), while PLZF and SALL4 are also expressed by larger chains of 8 and 16 A_al_ spermatogonia.

Similar observations of molecular heterogeneity among undifferentiated A_s_, A_pr_ and A_al_ spermatogonia have been reported in several recent studies (Tokuda *et al*. 2007; Grisanti *et al*. 2009; Sada *et al*. 2009; Suzuki *et al*. 2009; Zheng *et al*. 2009). The functional significance of this heterogeneity remains to be determined. Through the combination of FACS and MACS analyses, transplantation, genetic models and histochemical approaches, the phenotype of rodent SSCs is beginning to emerge. A list of putative SSC and undifferentiated spermatogonia markers is provided in table 1 along with the experimental evidence used to characterize each marker.

## 8. The spermatogonial stem cell niche

**Figure 5.**
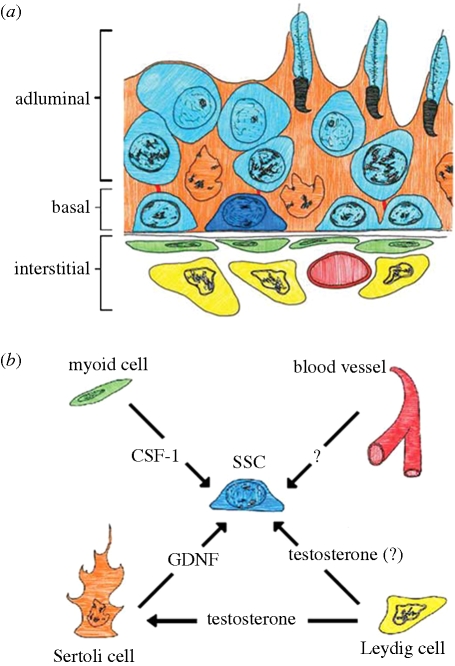
SSC niche. The SSC (dark blue) is diagrammed in its physical niche (*a*) surrounded by Sertoli cells (orange) and differentiating germ cells (light blue) within the seminiferous tubule. Niche components outside the tubule itself include myoid cells (green), blood vessels (red) and Leydig cells (yellow). The components of the niche and the some factors known to be provided by each are shown in (*b*). While some factors are known to act directly on the SSC, such as GDNF, others, like testosterone are important for spermatogenesis but may not act on the SSC.

SSCs reside within a specialized microenvironment called ‘niche’ that regulates testicular homeostasis by balancing SSC self-renewal and differentiation. A stem cell niche is comprised of cells, extracellular matrix components, and local soluble factors present in the vicinity of the stem cell that regulates cell fate. The structural basis for the SSC niche in the mammalian testis is the basal compartment of the seminiferous tubules that is composed of Sertoli cells and peritubular myoid cells (Dadoune 2007) (figure 5). Together, Sertoli and peritubular myoid cells secrete the basement membrane components to which the SSCs are connected via adhesion molecules (Tung *et al*. 1984). Sertoli cells are polarized columnar epithelial cells that support SSCs and differentiating germ cells by providing nutrients and mediating external signals in order to support spermatogenesis (Griswold 1998). The importance of Sertoli cells for germ cell differentiation is demonstrated by the transplantation of normal Sertoli cells into the testis of infertile mutant recipients with a Sertoli cell defect and successful initiation of spermatogenesis by recipient-derived spermatogonia (Kanatsu-Shinohara *et al*. 2003*b*, 2005*b*). Tight junctions between adjacent Sertoli cells constitute a protective blood–testis barrier (BTB) that divides the seminiferous epithelium into basal and adluminal compartments (figure 5*a*) and plays an important role in the regulation of germ cell differentiation (Cheng & Mruk 2002). The BTB maintains a selective substance flow between luminal fluid, blood plasma and interstitial fluid, thereby creating an immune-privileged environment for haploid germ cells in the adluminal compartment of the seminiferous tubules.

Along the length of the tubule, SSCs are thought to be localized in areas adjacent to the interstitial space (Chiarini-Garcia *et al*. 2003). Undifferentiated spermatogonia are observed predominantly in tubule areas adjacent to vasculature (Yoshida *et al*. 2007*b*; figure 5*a*).

The SSC niche mediates endocrine and paracrine signals that regulate self-renewal and differentiation (figure 5*b*). A key regulator of the SSC niche is GDNF which is secreted by Sertoli cells and acts through Ret receptor tyrosine kinase and GFRα1 co-receptor, which form a receptor complex on the surface of A_s_, A_Pr_ and A_al_ (Meng *et al*. 2000). Downstream signalling pathways that are activated by GDNF in undifferentiated spermatogonia are the PI3K/Akt pathway, members of the Src kinase family and the Ras/Erk1/2 pathway (Braydich-Stolle *et al*. 2007; Oatley *et al*. 2007; He *et al*. 2008). GDNF is thought to act through these pathways to regulate SSC self-renewal.

Targeted disruption of the Ets variant gene 5 (*Etv5*) results in defective maintenance of the SSC pool, whereas spermatogonial differentiation appears to be unaffected by this mutation (Chen *et al*. 2005). The transcription factor *Etv5* is expressed in Sertoli cells and loss of *Etv5* appears to impair the ability of Sertoli cells to support spermatogonia, possibly by disrupted BTB function as indicated by decreased Claudin-5 (CLDN5) levels in mutant mice (Morrow *et al*. 2009). In Sertoli cells, *Etv5* is upregulated by FGF2 *in vitro*, which is important for SSC renewal in culture (Kanatsu-Shinohara *et al*. 2003*a*; Kubota *et al*. 2004*a*; Yoon *et al*. 2009). Therefore, in addition to a direct effect of FGF2 on SSCs, an indirect paracrine effect of FGF2 on Sertoli cells appears possible.

The importance of peritubular myoid cells for spermatogonia maintenance has long been discussed. New data now suggest a role for the peritubular cell product colony-stimulating factor 1 (CSF1) on SSC maintenance (Oatley *et al*. 2009). *Csf1* was found to be expressed in interstitial Leydig and peritubular myoid cells, whereas the Csf1 receptor (*Csf1r*) was highly enriched in THY1^+^ cell fractions from pre-pubertal and adult mouse testis.

## 9. SSC culture

SSC culture provides a new approach for investigating the molecular mechanisms and cell-signalling pathways that regulate SSC function. While methods for maintaining and amplifying pluripotent ES and embryonic germ cells in culture are routine, methods for culturing adult tissue stem cells (including SSCs) had been more difficult to establish. However, tremendous progress culturing mouse and rat SSCs has been reported during the past 5–6 years (Kanatsu-Shinohara *et al*. 2003*a*; Kubota *et al*. 2004*a*,*b*; Hamra *et al*. 2005; Ryu *et al*. 2005). Rodent SSCs can now be maintained for a very long time (perhaps indefinitely) with a significant amplification in numbers. Stem cell activity in these cultures was confirmed by SSC transplantation, as diagrammatically represented in figure 2. The doubling time for mouse SSCs was determined to be 5.6 days (Kubota *et al*. 2004*b*), while the doubling time for rat SSCs is 3–4 days (Hamra *et al*. 2005) or 11 days (Ryu *et al*. 2005).

Several factors were critical to the establishment of long-term SSC cultures. First, methods to fractionate testis cell populations (FACS or MACS sorting and/or differential attachment and replating) resulted in the enrichment of SSCs and the removal of somatic cells that promote germ cell differentiation. Second, development of a serum-free, defined medium facilitated the discovery of essential growth factors. Specifically, GDNF is necessary to maintain and expand rodent SSCs in culture (Kanatsu-Shinohara *et al*. 2003*a*; Kubota *et al*. 2004*b*). The trophic effects of GDNF in both mice and rats is enhanced by the addition of soluble GFRα1 (the receptor for GDNF) and FGF2 (Kubota *et al*. 2004*a*; Ryu *et al*. 2005). Unlike mouse ES cells, the additions of leukaemia inhibitory factor (LIF) and foetal bovine serum (FBS) to cultures are superfluous and detrimental, respectively, in SSC cultures (Kubota *et al*. 2004*b*). Third, STO or mouse embryonic fibroblast (MEF) feeder cells are usually required. Whereas Shinohara's group has demonstrated that mouse SSCs can also be maintained in feeder-free conditions (Kanatsu-Shinohara *et al*. 2005*a*). SSC cultures are usually established from mouse pup testes (5–12 days postpartum) because SSCs are enriched at this stage of development. However, SSC cultures can be established from neonate (Kanatsu-Shinohara *et al*. 2003*a*) and adult mouse testis cells (Kubota *et al*. 2004*a*). Immortalized SSC lines have been established by the introduction of a retroviral telomerase gene (Feng *et al*. 2002) or treatment with the SV40 large T-antigen (Hofmann *et al*. 2005*a*). Evidence that each of these immortalized cell lines is spermatogonial-like is based primarily on genetic or immunocytochemical data, but transplantation data are lacking.

Stable SSC culture provides a valuable tool for dissecting mechanisms that regulate SSC renewal and differentiation. GDNF is required for SSC renewal *in vitro* (Kubota *et al*. 2004*a*) and *in vivo* (Meng *et al*. 2000). Through withdrawal and/or addition of GDNF to SSC cultures, two groups have now demonstrated that GDNF action is mediated by Src family kinases acting through PI3 kinase/Akt-dependent pathways (Braydich-Stolle *et al*. 2007; Oatley *et al*. 2007). In addition, microarray analysis identified genes that are regulated by GDNF withdrawal in SSC cultures. The importance of three of these genes (*Bcl6b*, *Erm* and *Lhx1*) was confirmed by transfecting SSC cultures with siRNAs specific for each gene. siRNA treatment caused decreased clump formation *in vitro* and decreased colonization of recipient testes after transplantation (Oatley *et al*. 2006, 2007).

Transfection of SSC cultures with siRNA, as described above, enables temporary knockdown of the target gene. To achieve stable knockdown of a target gene, short hairpin RNAs (shRNAs) can be coupled with lentiviral vectors. Dann *et al.* (2008) recently treated cultured SSCs with a lentiviral vector containing on *Oct-4*-targeted shRNA. The treatment caused a significant reduction in OCT-4 expression and reduced colonizing activity in the transplant assay by sixfold. Thus, through genetic manipulation and transplantation of SSC cultures, studies will continue to unravel regulatory pathways required for SSC self-renewal and differentiation.

## 10. Future directions

We have attempted to review the current state of knowledge and research in the biology of SSCs, focused primarily on the rodent model. Many areas of research are only beginning to be thoroughly investigated in SSCs, such as the molecular regulation of stem cell fate decisions and SSC heterogeneity. Recent progress characterizing, manipulating and culturing SSCs has opened the door to new experimental approaches for fundamental investigation and possible practical applications discussed below.

*In vitro* derivation of haploid gametes (elongated spermatids or sperm) may help to overcome spermatogenic barriers in infertile men. Feng *et al.* (2002) reported the production of spermatocytes and spermatids from a stable mouse SSC line, though the fertilization potential of these cells was not tested. Haploid male germ cells have also been generated by differentiation of ES cells in mice (Toyooka *et al*. 2003; Geijsen *et al*. 2004; Nayernia *et al*. 2006) and humans (Kee *et al*. 2009). Two studies demonstrated that *in vitro*, ESC-derived spermatids were competent to fertilize mouse eggs, generating blastocysts (Geijsen 2004) and live progeny (Nayernia *et al*. 2006), respectively. However, because of the epigenetic reprogramming that occurs during *in vivo* germ cell development, the epigenetic regulation of *in vitro* gametogenesis must be carefully assessed before clinical applications ensue (Georgiou *et al*. 2007). Generation of haploid germ cells from primary SSC cultures has not yet been reported, but this approach may have epigenetic advantages over ESC-derived gametes. Furthermore, progress establishing human SSC cultures will be an important experimental tool in a species where transplantation is not an option for characterizing SSCs.

In addition to trying to drive SSCs towards their typical biological end, there is evidence that SSCs are a source of pluripotent stem cells (Kanatsu-Shinohara *et al*. 2004*a*; Guan *et al*. 2006; Seandel *et al*. 2007). The ability to derive pluripotent stem cells from adult tissues, with the consent of the donor, may have some advantages over other approaches to pluripotency. However, more details are needed to understanding the genetic, epigenetic constitution of these cells, as well as their developmental potential.

Testicular tissues or testicular cell suspensions (containing SSCs) can be cryopreserved and may provide an avenue for preservation of valuable strains or species. Honaramooz *et al.* (2002) recently demonstrated that testicular tissues from newborn mice, pigs or goats can be grafted under the skin of immune-deficient mice and generate complete spermatogenesis. This approach has now been reported for several species (Oatley *et al*. 2004, 2005; Snedaker *et al*. 2004; Zeng *et al*. 2006; Kim *et al*. 2007; Arregui *et al*. 2008; Rodriguez-Sosa *et al*. 2010) and may allow germline preservation for endangered species or valuable domestic strains. Alternatively, valuable germlines can be preserved by freezing testis cell suspensions (containing SSCs) for future SSC transplantation. The proof in principle for this approach is already established for mice, rats, goats and dogs (Brinster & Avarbock 1994; Brinster *et al*. 2003; Honaramooz *et al*. 2003; Ryu *et al*. 2003; Kim *et al*. 2008).

SSC transplantation may have application for treating some cases of male infertility. For example, high-dose chemotherapy and total body radiation treatment of cancer can cause permanent infertility. While adult men can cryopreserve a semen sample prior to their oncologic treatment, this is not an option for pre-adolescent boys who are not yet making sperm. Using methods similar to those already established for other species, it may be possible for these young cancer patients to cryopreserve testis cells or tissue prior to cancer treatment and use those tissues to achieve fertility after they are cured (Orwig & Schlatt 2005; Goossens *et al*. 2008; Hermann *et al.* 2009). We have recently established a non-human primate model of cancer survivorship to test the safety and feasibility of SSC transplantation in a species that is relevant to human physiology (Hermann *et al*. 2007). Although SSC transplantation is not yet ready for the human fertility clinic, it may be reasonable for young cancer patients, with no other options to preserve their fertility, to cryopreserve testicular cells (Schlatt *et al*. 2009). Ginsberg and co-workers have been cryopreserving testicular tissue for young cancer patients since 2008 and report that this intervention is acceptable to parents and that testicular biopsies caused no acute adverse effects (Ginsberg *et al*. 2010). A human SSC culture system would be particularly useful in this setting because a few SSCs could be obtained in a small biopsy and expanded to a number sufficient for transplant therapy.

Progress studying SSC origins, regulation and activity over the past half century, has laid the foundation to pursue the clinical and veterinary options described in the preceding paragraphs. The field of SSC biology has grown substantially in the past two decades, fuelled in part by development of the SSC transplantation technique (Brinster & Avarbock 1994; Brinster & Zimmermann 1994), which impacted fundamental investigations as well as clinical application. Growth was also fuelled by the explosive development of the pluripotent stem cell and regenerative medicine fields. The next half century should bring many new discoveries about the biology and regenerative potential of SSCs that parallels the development of the haematopoietic stem cell field in the 1980s and 1990s.

## References

[RSTB20100026C1] Anderson R. Schaible K. Heasman J. Wylie C. 1999 Expression of the homophilic adhesion molecule, Ep-CAM, in the mammalian germ line. J. Reprod. Fertil. 116, 379–384 10615264 10.1530/jrf.0.1160379

[RSTB20100026C2] Antonangeli F. Giampietri C. Petrungaro S. Filippini A. Ziparo E. 2009 Expression profile of a 400-bp Stra8 promoter region during spermatogenesis. Microsc. Res. Tech. 72, 816–822 19378319 10.1002/jemt.20724

[RSTB20100026C3] Arregui L. Rathi R. Megee S. O. Honaramooz A. Gomendio M. Roldan E. R. Dobrinski I. 2008 Xenografting of sheep testis tissue and isolated cells as a model for preservation of genetic material from endangered ungulates. Reproduction 136, 85–93 (doi:10.1530/REP-07-0433) 18390693 10.1530/REP-07-0433

[RSTB20100026C4] Ballow D. Meistrich M. L. Matzuk M. Rajkovic A. 2006 *a* Sohlh1 is essential for spermatogonial differentiation. Dev. Biol. 294, 161–167 (doi:10.1016/j.ydbio.2006.02.027) 16564520 10.1016/j.ydbio.2006.02.027

[RSTB20100026C5] Ballow D. J. Xin Y. Choi Y. Pangas S. A. Rajkovic A. 2006 *b* Sohlh2 is a germ cell-specific bHLH transcription factor. Gene Expr. Patterns 6, 1014–1018 (doi:10.1016/j.modgep.2006.04.007) 16765102 10.1016/j.modgep.2006.04.007

[RSTB20100026C6] Bendel-Stenzel M. Anderson R. Heasman J. Wylie C. 1998 The origin and migration of primordial germ cells in the mouse. Semin. Cell Dev. Biol. 9, 393–400 9813186 10.1006/scdb.1998.0204

[RSTB20100026C7] Braun R. E. Behringer R. R. Peschon J. J. Brinster R. L. Palmiter R. D. 1989 Genetically haploid spermatids are phenotypically diploid. Nature 337, 373–376 (doi:10.1038/337373a0) 2911388 10.1038/337373a0

[RSTB20100026C8] Braydich-Stolle L. Kostereva N. Dym M. Hofmann M. C. 2007 Role of Src family kinases and N-Myc in spermatogonial stem cell proliferation. Dev. Biol. 304, 34–45 17222400 10.1016/j.ydbio.2006.12.013 PMC2077853

[RSTB20100026C9] Brinster R. L. Avarbock M. R. 1994 Germline transmission of donor haplotype following spermatogonial transplantation. Proc. Natl Acad. Sci. USA 91, 11 303–11 307 (doi:10.1073/pnas.91.24.11303) 7972054 10.1073/pnas.91.24.11303 PMC45219

[RSTB20100026C10] Brinster R. L. Zimmermann J. W. 1994 Spermatogenesis following male germ-cell transplantation. Proc. Natl Acad. Sci. USA 91, 11 298–11 302 (doi:10.1073/pnas.91.24.11298) 7972053 10.1073/pnas.91.24.11298 PMC45218

[RSTB20100026C11] Brinster C. J. Ryu B. Y. Avarbock M. R. Karagenc L. Brinster R. L. Orwig K. E. 2003 Restoration of fertility by germ cell transplantation requires effective recipient preparation. Biol. Reprod. 69, 412–420 (doi:10.1095/biolreprod.103.016519) 12672656 10.1095/biolreprod.103.016519

[RSTB20100026C12] Buaas F. W. Kirsh A. L. Sharma M. McLean D. J. Morris J. L. Griswold M. D. de Rooij D. G. Braun R. E. 2004 Plzf is required in adult male germ cells for stem cell self-renewal. Nat. Genet. 36, 647–652 (doi:10.1038/ng1366) 15156142 10.1038/ng1366

[RSTB20100026C13] Buageaw A. Sukhwani M. Ben-Yehudah A. Ehmcke J. Rawe V. Y. Pholpramool C. Orwig K. E. Schlatt S. 2005 GDNF family receptor alpha1 phenotype of spermatogonial stem cells in immature mouse testes. Biol. Reprod. 73, 1011–1016 (doi:10.1095/biolreprod.105.043810) 16014811 10.1095/biolreprod.105.043810

[RSTB20100026C14] Chen C. 2005 ERM is required for transcriptional control of the spermatogonial stem cell niche. Nature 436, 1030–1034 (doi:10.1038/nature03894) 16107850 10.1038/nature03894 PMC2909764

[RSTB20100026C15] Cheng C. Y. Mruk D. D. 2002 Cell junction dynamics in the testis: sertoli–germ cell interactions and male contraceptive development. Physiol. Rev. 82, 825–874 12270945 10.1152/physrev.00009.2002

[RSTB20100026C16] Chiarini-Garcia H. Raymer A. M. Russell L. D. 2003 Non-random distribution of spermatogonia in rats: evidence of niches in the seminiferous tubules. Reproduction 126, 669–680 (doi:10.1530/rep.0.1260669) 14611641 10.1530/rep.0.1260669

[RSTB20100026C17] Clermont Y. 1972 Kinetics of spermatogenesis in mammals: seminiferous epithelium cycle and spermatogonial renewal. Physiol. Rev. 52, 198–236 4621362 10.1152/physrev.1972.52.1.198

[RSTB20100026C18] Clermont Y. Antar M. 1973 Duration of the cycle of the seminiferous epithelium and the spermatogonial renewal in the monkey, *Macaca arctoides*. Am. J. Anat. 136, 153–165 (doi:10.1002/aja.1001360204) 4684877 10.1002/aja.1001360204

[RSTB20100026C19] Clermont Y. Bustos-Obregon E. 1968 Re-examination of spermatogonial renewal in the rat by means of seminiferous tubules mounted ‘in toto’. Am. J. Anat. 122, 237–247 (doi:10.1002/aja.1001220205) 5659131 10.1002/aja.1001220205

[RSTB20100026C20] Clermont Y. Hermo L. 1975 Spermatogonial stem cells in the albino rat. Am. J. Anat. 142, 159–175 (doi:10.1002/aja.1001420203) 1115004 10.1002/aja.1001420203

[RSTB20100026C21] Clermont Y. Leblond C. P. 1953 Renewal of spermatogonia in the rat. Am. J. Anat. 93, 475–501 (doi:10.1002/aja.1000930308) 13104341 10.1002/aja.1000930308

[RSTB20100026C22] Clermont Y. Leblond C. P. 1959 Differentiation and renewal of spermatogonia in the monkey, *Macacus rhesus*. Am. J. Anat. 104, 237–273 (doi:10.1002/aja.1001040204) 13810669 10.1002/aja.1001040204

[RSTB20100026C23] Clermont Y. Perey B. 1957 Quantitative study of the cell population of the seminiferous tubules in immature rats. Am. J. Anat. 100, 241–267 (doi:10.1002/aja.1001000205) 13435229 10.1002/aja.1001000205

[RSTB20100026C24] Cooke H. J. Lee M. Kerr S. Ruggiu M. 1996 A murine homologue of the human DAZ gene is autosomal and expressed only in male and female gonads. Hum. Mol. Genet. 5, 513–516 (doi:10.1093/hmg/5.4.513) 8845845 10.1093/hmg/5.4.513

[RSTB20100026C25] Corallini S. Fera S. Grisanti L. Falciatori I. Muciaccia B. Stefanini M. Vicini E. 2006 Expression of the adaptor protein m-Numb in mouse male germ cells. Reproduction 132, 887–897 (doi:10.1530/REP-06-0062) 17127749 10.1530/REP-06-0062

[RSTB20100026C26] Costoya J. A. Hobbs R. M. Barna M. Cattoretti G. Manova K. Sukhwani M. Orwig K. E. Wolgemuth D. J. Pandolfi P. P. 2004 Essential role of Plzf in maintenance of spermatogonial stem cells. Nat. Genet. 36, 653–659 (doi:10.1038/ng1367) 15156143 10.1038/ng1367

[RSTB20100026C27] Dadoune J. P. 2007 New insights into male gametogenesis: what about the spermatogonial stem cell niche? Folia Histochem. Cytobiol. 45, 141–147 17951161

[RSTB20100026C28] Dann C. T. Alvarado A. L. Molyneux L. A. Denard B. S. Garbers D. L. Porteus M. H. 2008 Spermatogonial stem cell self-renewal requires OCT4, a factor downregulated during retinoic acid-induced differentiation. Stem Cells 26, 2928–2937 (doi:10.1634/stemcells.2008-0134) 18719224 10.1634/stemcells.2008-0134

[RSTB20100026C29] Dettin L. Ravindranath N. Hofmann M. C. Dym M. 2003 Morphological characterization of the spermatogonial subtypes in the neonatal mouse testis. Biol. Reprod. 69, 1565–1571 (doi:10.1095/biolreprod.103.016394) 12855601 10.1095/biolreprod.103.016394

[RSTB20100026C30] Dobrinski I. Ogawa T. Avarbock M. R. Brinster R. L. 1999 Computer assisted image analysis to assess colonization of recipient seminiferous tubules by spermatogonial stem cells from transgenic donor mice. Mol. Reprod. Dev. 53, 142–148 (doi:10.1002/(SICI)1098-2795(199906)53:2<142::AID-MRD3>3.0.CO;2-O) 10331452 10.1002/(SICI)1098-2795(199906)53:2<142::AID-MRD3>3.0.CO;2-O

[RSTB20100026C31] Dym M. Clermont Y. 1970 Role of spermatogonia in the repair of the seminiferous epithelium following x-irradiation of the rat testis. Am. J. Anat. 128, 265–282 (doi:10.1002/aja.1001280302) 4193812 10.1002/aja.1001280302

[RSTB20100026C32] Ebata K. T. Zhang X. Nagano M. C. 2005 Expression patterns of cell-surface molecules on male germ line stem cells during postnatal mouse development. Mol. Reprod. Dev. 72, 171–181 (doi:10.1002/mrd.20324) 16010662 10.1002/mrd.20324

[RSTB20100026C33] Elling U. Klasen C. Eisenberger T. Anlag K. Treier M. 2006 Murine inner cell mass-derived lineages depend on Sall4 function. Proc. Natl Acad. Sci. USA 103, 16 319–16 324 (doi:10.1073/pnas.0607884103) 10.1073/pnas.0607884103 PMC1637580 17060609

[RSTB20100026C34] Enders G. C. May J. J. 2nd 1994 Developmentally regulated expression of a mouse germ cell nuclear antigen examined from embryonic day 11 to adult in male and female mice. Dev. Biol. 163, 331–340 (doi:10.1006/dbio.1994.1152) 8200475 10.1006/dbio.1994.1152

[RSTB20100026C35] Falender A. E. Freiman R. N. Geles K. G. Lo K. C. Hwang K. Lamb D. J. Morris P. L. Tjian R. Richards J. S. 2005 Maintenance of spermatogenesis requires TAF4b, a gonad-specific subunit of TFIID. Genes Dev. 19, 794–803 (doi:10.1101/gad.1290105) 15774719 10.1101/gad.1290105 PMC1074317

[RSTB20100026C36] Feng L. X. Chen Y. Dettin L. Pera R. A. Herr J. C. Goldberg E. Dym M. 2002 Generation and *in vitro* differentiation of a spermatogonial cell line. Science 297, 392–395 (doi:10.1126/science.1073162) 12077424 10.1126/science.1073162

[RSTB20100026C159] Filipponi D. Hobbs R. M. Ottolenghi S. Rossi P. Jannini E. A. Pandolfi P. P. Dolci S. 2007 Repression of kit expression by Plzf in germ cells. Mol. Cell. Biol. 27, 6770–6781 (doi:10.1128/MCB.00479-07) 17664282 10.1128/MCB.00479-07 PMC2099235

[RSTB20100026C37] Fujita K. Ohta H. Tsujimura A. Takao T. Miyagawa Y. Takada S. Matsumiya K. Wakayama T. Okuyama A. 2005 Transplantation of spermatogonial stem cells isolated from leukemic mice restores fertility without inducing leukemia. J. Clin. Invest. 115, 1855–1861 (doi:10.1172/JCI24189) 15965502 10.1172/JCI24189 PMC1150287

[RSTB20100026C38] Fujiwara Y. Komiya T. Kawabata H. Sato M. Fujimoto H. Furusawa M. Noce T. 1994 Isolation of a DEAD-family protein gene that encodes a murine homolog of *Drosophila vasa* and its specific expression in germ cell lineage. Proc. Natl Acad. Sci. USA 91, 12 258–12 262 (doi:10.1073/pnas.91.25.12258) 10.1073/pnas.91.25.12258 PMC45416 7991615

[RSTB20100026C39] Geijsen N. Horoschak M. Kim K. Gribnau J. Eggan K. Daley G. Q. 2004 Derivation of embryonic germ cells and male gametes from embryonic stem cells. Nature 427, 148–154 (doi:10.1038/nature02247) 14668819 10.1038/nature02247

[RSTB20100026C40] Georgiou I. 2007 *In vitro* spermatogenesis as a method to bypass pre-meiotic or post-meiotic barriers blocking the spermatogenetic process: genetic and epigenetic implications in assisted reproductive technology. Andrologia 39, 159–176 (doi:10.1111/j.1439-0272.2007.00778.x) 17714214 10.1111/j.1439-0272.2007.00778.x

[RSTB20100026C41] Ginsberg J. P. Carlson C. A. Lin K. Hobbie W. L. Wigo E. Wu X. Brinster R. L. Kolon T. F. 2010 An experimental protocol for fertility preservation in prepubertal boys recently diagnosed with cancer: a report of acceptability and safety. Hum. Reprod. 25, 37–41 (doi:10.1093/humrep/dep371) 19861330 10.1093/humrep/dep371 PMC2794668

[RSTB20100026C42] Ginsburg M. Snow M. H. McLaren A. 1990 Primordial germ cells in the mouse embryo during gastrulation. Development 110, 521–528 2133553 10.1242/dev.110.2.521

[RSTB20100026C43] Giuili G. Tomljenovic A. Labrecque N. Oulad-Abdelghani M. Rassoulzadegan M. Cuzin F. 2002 Murine spermatogonial stem cells: targeted transgene expression and purification in an active state. EMBO Rep. 3, 753–759 (doi:10.1093/embo-reports/kvf149) 12151334 10.1093/embo-reports/kvf149 PMC1084203

[RSTB20100026C44] Goossens E. Geens M. De Block G. Tournaye H. 2008 Spermatogonial survival in long-term human prepubertal xenografts. Fertil. Steril. 90, 2019–2022 (doi:10.1016/j.fertnstert.2007.09.044) 18439593 10.1016/j.fertnstert.2007.09.044

[RSTB20100026C45] Grisanti L. 2009 Identification of spermatogonial stem cell subsets by morphological analysis and prospective isolation. Stem Cells 27, 3043–3052 19711452 10.1002/stem.206

[RSTB20100026C46] Griswold M. D. 1998 The central role of Sertoli cells in spermatogenesis. Semin. Cell Dev. Biol. 9, 411–416 (doi:10.1006/scdb.1998.0203) 9813187 10.1006/scdb.1998.0203

[RSTB20100026C47] Guan K. 2006 Pluripotency of spermatogonial stem cells from adult mouse testis. Nature 440, 1199–1203 (doi:10.1038/nature04697) 16565704 10.1038/nature04697

[RSTB20100026C48] Hamra F. K. Schultz N. Chapman K. M. Grellhesl D. M. Cronkhite J. T. Hammer R. E. Garbers D. L. 2004 Defining the spermatogonial stem cell. Dev. Biol. 269, 393–410 15110708 10.1016/j.ydbio.2004.01.027

[RSTB20100026C49] Hamra F. K. Chapman K. M. Nguyen D. M. Williams-Stephens A. A. Hammer R. E. Garbers D. L. 2005 Self renewal, expansion, and transfection of rat spermatogonial stem cells in culture. Proc. Natl Acad. Sci. USA 102, 17 430–17 435 (doi:10.1073/pnas.0508780102) 10.1073/pnas.0508780102 PMC1283987 16293688

[RSTB20100026C50] He Z. Jiang J. Kokkinaki M. Golestaneh N. Hofmann M. C. Dym M. 2008 Gdnf upregulates c-Fos transcription via the Ras/Erk1/2 pathway to promote mouse spermatogonial stem cell proliferation. Stem Cells 26, 266–278 (doi:10.1634/stemcells.2007-0436) 17962702 10.1634/stemcells.2007-0436 PMC2905627

[RSTB20100026C51] Hermann B. P. 2007 Characterization, cryopreservation and ablation of spermatogonial stem cells in adult rhesus macaques. Stem Cells 25, 2330–2338 (doi:10.1634/stemcells.2007-0143) 17585169 10.1634/stemcells.2007-0143 PMC3593092

[RSTB20100026C160] Hermann B. M. Sukhwani M. Hansel M. Orwig K. 2009 Spermatogonial stem cells in higher primates: are there differences to those in rodents? Reproduction 139, 479–493 (doi:10.1530/REP-09-0255) 19880674 10.1530/REP-09-0255 PMC2895987

[RSTB20100026C52] Hilscher B. Hilscher W. Maurer W. 1969 Autoradiographic studies on the modus of proliferation and regeneration of the seminiferous epithelium of Wistar rats. Z. Zellforsch. Mikrosk. Anat. 94, 593–604 (doi:10.1007/BF00936064) 5809191

[RSTB20100026C53] Hofmann M. C. Braydich-Stolle L. Dettin L. Johnson E. Dym M. 2005 *a* Immortalization of mouse germ line stem cells. Stem Cells 23, 200–210 (doi:10.1634/stemcells.2003-0036) 15671143 10.1634/stemcells.2003-0036 PMC3151429

[RSTB20100026C54] Hofmann M. C. Braydich-Stolle L. Dym M. 2005 *b* Isolation of male germ-line stem cells; influence of GDNF. Dev. Biol. 279, 114–124 (doi:10.1016/j.ydbio.2004.12.006) 15708562 10.1016/j.ydbio.2004.12.006 PMC2904978

[RSTB20100026C55] Honaramooz A. Snedaker A. Boiani M. Scholer H. Dobrinski I. Schlatt S. 2002 Sperm from neonatal mammalian testes grafted in mice. Nature 418, 778–781 (doi:10.1038/nature00918) 12181567 10.1038/nature00918

[RSTB20100026C56] Honaramooz A. Behboodi E. Megee S. O. Overton S. A. Galantino-Homer H. Echelard Y. Dobrinski I. 2003 Fertility and germline transmission of donor haplotype following germ cell transplantation in immunocompetent goats. Biol. Reprod. 69, 1260–1264 (doi:10.1095/biolreprod.103.018788) 12801978 10.1095/biolreprod.103.018788

[RSTB20100026C57] Huckins C. 1971 *a* The spermatogonial stem cell population in adult rats. II. A radioautographic analysis of their cell cycle properties. Cell Tissue Kinet. 4, 313–334 5127356 10.1111/j.1365-2184.1971.tb01543.x

[RSTB20100026C58] Huckins C. 1971 *b* Cell cycle properties of differentiating spermatogonia in adult Sprague–Dawley rats. Cell Tissue Kinet. 4, 139–154 5128278 10.1111/j.1365-2184.1971.tb01524.x

[RSTB20100026C59] Huckins C. 1971 *c* The spermatogonial stem cell population in adult rats. I. Their morphology, proliferation and maturation. Anat. Rec. 169, 533–557 (doi:10.1002/ar.1091690306) 5550532 10.1002/ar.1091690306

[RSTB20100026C60] Jarvis S. Elliott D. J. Morgan D. Winston R. Readhead C. 2005 Molecular markers for the assessment of postnatal male germ cell development in the mouse. Hum. Reprod. 20, 108–116 (doi:10.1093/humrep/deh565) 15539445 10.1093/humrep/deh565

[RSTB20100026C61] Kanatsu-Shinohara M. Ogonuki N. Inoue K. Miki H. Ogura A. Toyokuni S. Shinohara T. 2003 *a* Long-term proliferation in culture and germline transmission of mouse male germline stem cells. Biol. Reprod. 69, 612–616 (doi:10.1095/biolreprod.103.017012) 12700182 10.1095/biolreprod.103.017012

[RSTB20100026C62] Kanatsu-Shinohara M. Ogonuki N. Inoue K. Ogura A. Toyokuni S. Shinohara T. 2003 *b* Restoration of fertility in infertile mice by transplantation of cryopreserved male germline stem cells. Hum. Reprod. 18, 2660–2667 (doi:10.1093/humrep/deg483) 14645188 10.1093/humrep/deg483

[RSTB20100026C63] Kanatsu-Shinohara M. 2004 *a* Generation of pluripotent stem cells from neonatal mouse testis. Cell 119, 1001–1012 (doi:10.1016/j.cell.2004.11.011) 15620358 10.1016/j.cell.2004.11.011

[RSTB20100026C64] Kanatsu-Shinohara M. Toyokuni S. Shinohara T. 2004 *b* CD9 is a surface marker on mouse and rat male germline stem cells. Biol. Reprod. 70, 70–75 (doi:10.1095/biolreprod.103.020867) 12954725 10.1095/biolreprod.103.020867

[RSTB20100026C65] Kanatsu-Shinohara M. Miki H. Inoue K. Ogonuki N. Toyokuni S. Ogura A. Shinohara T. 2005 *a* Long-term culture of mouse male germline stem cells under serum- or feeder-free conditions. Biol. Reprod. 72, 985–991 15601913 10.1095/biolreprod.104.036400

[RSTB20100026C66] Kanatsu-Shinohara M. Miki H. Inoue K. Ogonuki N. Toyokuni S. Ogura A. Shinohara T. 2005 *b* Germline niche transplantation restores fertility in infertile mice. Hum. Reprod. 20, 2376–2382 15919776 10.1093/humrep/dei096

[RSTB20100026C67] Kanatsu-Shinohara M. Inoue K. Miki H. Ogonuki N. Takehashi M. Morimoto T. Ogura A. Shinohara T. 2006 Clonal origin of germ cell colonies after spermatogonial transplantation in mice. Biol. Reprod. 75, 68–74 (doi:10.1095/biolreprod.106.051193) 16598026 10.1095/biolreprod.106.051193

[RSTB20100026C68] Kanatsu-Shinohara M. 2008 Homing of mouse spermatogonial stem cells to germline niche depends on beta1-integrin. Cell Stem Cell 3, 533–542 18983968 10.1016/j.stem.2008.08.002

[RSTB20100026C69] Kee K. Angeles V. T. Flores M. Nguyen H. N. Reijo Pera R. A. 2009 Human DAZL, DAZ and BOULE genes modulate primordial germ-cell and haploid gamete formation. Nature 462, 222–225 (doi:10.1038/nature08562) 19865085 10.1038/nature08562 PMC3133736

[RSTB20100026C70] Kim Y. Selvaraj V. Pukazhenthi B. Travis A. J. 2007 Effect of donor age on success of spermatogenesis in feline testis xenografts. Reprod. Fertil. Dev. 19, 869–876 (doi:10.1071/RD07056) 17897590 10.1071/rd07056

[RSTB20100026C71] Kim Y. Turner D. Nelson J. Dobrinski I. McEntee M. Travis A. J. 2008 Production of donor-derived sperm after spermatogonial stem cell transplantation in the dog. Reproduction 136, 823–831 (doi:10.1530/REP-08-0226) 18768666 10.1530/REP-08-0226 PMC2706094

[RSTB20100026C72] Kluin P. M. de Rooij D. G. 1981 A comparison between the morphology and cell kinetics of gonocytes and adult type undifferentiated spermatogonia in the mouse. Int. J. Androl. 4, 475–493 (doi:10.1111/j.1365-2605.1981.tb00732.x) 7298230 10.1111/j.1365-2605.1981.tb00732.x

[RSTB20100026C73] Koshimizu U. Nishioka H. Watanabe D. Dohmae K. Nishimune Y. 1995 Characterization of a novel spermatogenic cell antigen specific for early stages of germ cells in mouse testis. Mol. Reprod. Dev. 40, 221–227 (doi:10.1002/mrd.1080400211) 7766415 10.1002/mrd.1080400211

[RSTB20100026C74] Kubota H. Avarbock M. R. Brinster R. L. 2003 Spermatogonial stem cells share some, but not all, phenotypic and functional characteristics with other stem cells. Proc. Natl Acad. Sci. USA 100, 6487–6492 (doi:10.1073/pnas.0631767100) 12738887 10.1073/pnas.0631767100 PMC164473

[RSTB20100026C75] Kubota H. Avarbock M. R. Brinster R. L. 2004 *a* Growth factors essential for self-renewal and expansion of mouse spermatogonial stem cells. Proc. Natl Acad. Sci. USA 101, 16 489–16 494 (doi:10.1073/pnas.0407063101) 15520394 10.1073/pnas.0407063101 PMC534530

[RSTB20100026C76] Kubota H. Avarbock M. R. Brinster R. L. 2004 *b* Culture conditions and single growth factors affect fate determination of mouse spermatogonial stem cells. Biol. Reprod. 71, 722–731 (doi:10.1095/biolreprod.104.029207) 15115718 10.1095/biolreprod.104.029207

[RSTB20100026C77] Lawson K. A. Dunn N. R. Roelen B. A. Zeinstra L. M. Davis A. M. Wright C. V. Korving J. P. Hogan B. L. 1999 Bmp4 is required for the generation of primordial germ cells in the mouse embryo. Genes Dev. 13, 424–436 (doi:10.1101/gad.13.4.424) 10049358 10.1101/gad.13.4.424 PMC316469

[RSTB20100026C78] Leblond C. P. Clermont Y. 1952 *a* Spermiogenesis of rat, mouse, hamster and guinea pig as revealed by the periodic acid-fuchsin sulfurous acid technique. Am. J. Anat. 90, 167–215 (doi:10.1002/aja.1000900202) 14923625 10.1002/aja.1000900202

[RSTB20100026C79] Leblond C. P. Clermont Y. 1952 *b* Definition of the stages of the cycle of the seminiferous epithelium in the rat. Ann. N.Y. Acad. Sci. 55, 548–573 (doi:10.1111/j.1749-6632.1952.tb26576.x) 13139144 10.1111/j.1749-6632.1952.tb26576.x

[RSTB20100026C80] Lim C. Y. 2008 Sall4 regulates distinct transcription circuitries in different blastocyst-derived stem cell lineages. Cell Stem Cell 3, 543–554 (doi:10.1016/j.stem.2008.08.004) 18804426 10.1016/j.stem.2008.08.004

[RSTB20100026C81] Lo K. C. Brugh V. M. 3rd Parker M. Lamb D. J. 2005 Isolation and enrichment of murine spermatogonial stem cells using rhodamine 123 mitochondrial dye. Biol. Reprod. 72, 767–771 (doi:10.1095/biolreprod.104.033464) 15576830 10.1095/biolreprod.104.033464

[RSTB20100026C82] Lolicato F. Marino R. Paronetto M. P. Pellegrini M. Dolci S. Geremia R. Grimaldi P. 2008 Potential role of Nanos3 in maintaining the undifferentiated spermatogonia population. Dev. Biol. 313, 725–738 (doi:10.1016/j.ydbio.2007.11.011) 18089289 10.1016/j.ydbio.2007.11.011

[RSTB20100026C83] Manova K. Nocka K. Besmer P. Bachvarova R. F. 1990 Gonadal expression of c-kit encoded at the W locus of the mouse. Development 110, 1057–1069 1712701 10.1242/dev.110.4.1057

[RSTB20100026C84] McCarrey J. 1993 Development of the germ cell. In Cell and molecular biology of the testis (eds Desjardins C. Ewing L.), pp. 58–89 New York, NY: Oxford University Press

[RSTB20100026C85] McLaren A. 2003 Primordial germ cells in the mouse. Dev. Biol. 262, 1–15 (doi:10.1016/S0012-1606(03)00214-8) 14512014 10.1016/s0012-1606(03)00214-8

[RSTB20100026C86] Meng X. 2000 Regulation of cell fate decision of undifferentiated spermatogonia by GDNF. Science 287, 1489–1493 (doi:10.1126/science.287.5457.1489) 10688798 10.1126/science.287.5457.1489

[RSTB20100026C87] Monesi V. 1962 Autoradiographic study of DNA synthesis and the cell cycle in spermatogonia and spermatocytes of mouse testis using tritiated thymidine. J. Cell Biol. 14, 1–18 (doi:10.1083/jcb.14.1.1) 14475361 10.1083/jcb.14.1.1 PMC2106095

[RSTB20100026C88] Morimoto H. Kanatsu-Shinohara M. Takashima S. Chuma S. Nakatsuji N. Takehashi M. Shinohara T. 2009 Phenotypic plasticity of mouse spermatogonial stem cells. PLoS One 4, e7909 (doi:10.1371/journal.pone.0007909) 19936070 10.1371/journal.pone.0007909 PMC2774941

[RSTB20100026C89] Morrow C. M. Tyagi G. Simon L. Carnes K. Murphy K. M. Cooke P. S. Hofmann M. C. Hess R. A. 2009 Claudin 5 expression in mouse seminiferous epithelium is dependent upon the transcription factor Ets-variant 5 and contributes to blood–testis barrier function. Biol. Reprod. 81, 871–879 19571261 10.1095/biolreprod.109.077040 PMC2770019

[RSTB20100026C90] Nakagawa T. Nabeshima Y. Yoshida S. 2007 Functional identification of the actual and potential stem cell compartments in mouse spermatogenesis. Dev. Cell 12, 195–206 (doi:10.1016/j.devcel.2007.01.002) 17276338 10.1016/j.devcel.2007.01.002

[RSTB20100026C91] Naughton C. K. Jain S. Strickland A. M. Gupta A. Milbrandt J. 2006 Glial cell-line derived neurotrophic factor-mediated RET signaling regulates spermatogonial stem cell fate. Biol. Reprod. 74, 314–321 (doi:10.1095/biolreprod.105.047365) 16237148 10.1095/biolreprod.105.047365

[RSTB20100026C92] Nayernia K. 2006 *In vitro*-differentiated embryonic stem cells give rise to male gametes that can generate offspring mice. Dev. Cell 11, 125–132 (doi:10.1016/j.devcel.2006.05.010) 16824959 10.1016/j.devcel.2006.05.010

[RSTB20100026C93] Niederberger C. Agulnik A. I. Cho Y. Lamb D. Bishop C. E. 1997 *In situ* hybridization shows that Dazla expression in mouse testis is restricted to premeiotic stages IV–VI of spermatogenesis. Mamm. Genome 8, 277–278 (doi:10.1007/s003359900409) 9096110 10.1007/s003359900409

[RSTB20100026C94] Oakberg E. F. 1956 *a* A description of spermiogenesis in the mouse and its use in analysis of the cycle of the seminiferous epithelium and germ cell renewal. Am. J. Anat. 99, 391–413 (doi:10.1002/aja.1000990303) 13402725 10.1002/aja.1000990303

[RSTB20100026C95] Oakberg E. F. 1956 *b* Duration of spermatogenesis in the mouse and timing of stages of the cycle of the seminiferous epithelium. Am. J. Anat. 99, 507–516 (doi:10.1002/aja.1000990307) 13402729 10.1002/aja.1000990307

[RSTB20100026C96] Oakberg E. F. 1971 Spermatogonial stem-cell renewal in the mouse. Anat. Rec. 169, 515–531 (doi:10.1002/ar.1091690305) 5550531 10.1002/ar.1091690305

[RSTB20100026C97] Oatley J. M. de Avila D. M. Reeves J. J. McLean D. J. 2004 Spermatogenesis and germ cell transgene expression in xenografted bovine testicular tissue. Biol. Reprod. 71, 494–501 (doi:10.1095/biolreprod.104.027953) 15070832 10.1095/biolreprod.104.027953

[RSTB20100026C98] Oatley J. M. Reeves J. J. McLean D. J. 2005 Establishment of spermatogenesis in neonatal bovine testicular tissue following ectopic xenografting varies with donor age. Biol. Reprod. 72, 358–364 (doi:10.1095/biolreprod.104.030783) 15470000 10.1095/biolreprod.104.030783

[RSTB20100026C99] Oatley J. M. Avarbock M. R. Telaranta A. I. Fearon D. T. Brinster R. L. 2006 Identifying genes important for spermatogonial stem cell self-renewal and survival. Proc. Natl Acad. Sci. USA 103, 9524–9529 (doi:10.1073/pnas.0603332103) 16740658 10.1073/pnas.0603332103 PMC1480440

[RSTB20100026C100] Oatley J. M. Avarbock M. R. Brinster R. L. 2007 Glial cell line-derived neurotrophic factor regulation of genes essential for self-renewal of mouse spermatogonial stem cells is dependent on SRC family kinase signaling. J. Biol. Chem. 282, 25 842–25 851 10.1074/jbc.M703474200 PMC4083858 17597063

[RSTB20100026C101] Oatley J. M. Oatley M. J. Avarbock M. R. Tobias J. W. Brinster R. L. 2009 Colony stimulating factor 1 is an extrinsic stimulator of mouse spermatogonial stem cell self-renewal. Development 136, 1191–1199 (doi:10.1242/dev.032243) 19270176 10.1242/dev.032243 PMC2685936

[RSTB20100026C102] Ogawa T. Dobrinski I. Avarbock M. R. Brinster R. L. 2000 Transplantation of male germ line stem cells restores fertility in infertile mice. Nat. Med. 6, 29–34 10613820 10.1038/71496 PMC4879876

[RSTB20100026C103] Ohbo K. 2003 Identification and characterization of stem cells in prepubertal spermatogenesis in mice. Dev. Biol. 258, 209–225 (doi:10.1016/S0012-1606(03)00111-8) 12781694 10.1016/s0012-1606(03)00111-8

[RSTB20100026C104] Ohmura M. Yoshida S. Ide Y. Nagamatsu G. Suda T. Ohbo K. 2004 Spatial analysis of germ stem cell development in Oct-4/EGFP transgenic mice. Arch. Histol. Cytol. 67, 285–296 (doi:10.1679/aohc.67.285) 15700536 10.1679/aohc.67.285

[RSTB20100026C105] Ohmura M. 2008 Identification of stem cells during prepubertal spermatogenesis via monitoring of nucleostemin promoter activity. Stem Cells 26, 3237–3246 18802033 10.1634/stemcells.2008-0506

[RSTB20100026C106] Orwig K. E. Schlatt S. 2005 Cryopreservation and transplantation of spermatogonia and testicular tissue for preservation of male fertility. J. Natl Cancer Inst. Monogr. 2005, 51–56 (doi:10.1093/jncimonographs/lgi029) 15784824 10.1093/jncimonographs/lgi029

[RSTB20100026C107] Osawa M. Hanada K. Hamada H. Nakauchi H. 1996 Long-term lymphohematopoietic reconstitution by a single CD34-low/negative hematopoietic stem cell. Science 273, 242–245 (doi:10.1126/science.273.5272.242) 8662508 10.1126/science.273.5272.242

[RSTB20100026C108] Oulad-Abdelghani M. Bouillet P. Decimo D. Gansmuller A. Heyberger S. Dolle P. Bronner S. Lutz Y. Chambon P. 1996 Characterization of a premeiotic germ cell-specific cytoplasmic protein encoded by Stra8, a novel retinoic acid-responsive gene. J. Cell Biol. 135, 469–477 (doi:10.1083/jcb.135.2.469) 8896602 10.1083/jcb.135.2.469 PMC2121034

[RSTB20100026C109] Pesce M. Wang X. Wolgemuth D. J. Scholer H. 1998 Differential expression of the Oct-4 transcription factor during mouse germ cell differentiation. Mech. Dev. 71, 89–98 (doi:10.1016/S0925-4773(98)00002-1) 9507072 10.1016/s0925-4773(98)00002-1

[RSTB20100026C110] Raverot G. Weiss J. Park S. Y. Hurley L. Jameson J. L. 2005 Sox3 expression in undifferentiated spermatogonia is required for the progression of spermatogenesis. Dev. Biol. 283, 215–225 (doi:10.1016/j.ydbio.2005.04.013) 15893302 10.1016/j.ydbio.2005.04.013

[RSTB20100026C111] Reid A. 1995 Leukemia translocation gene, PLZF, is expressed with a speckled nuclear pattern in early hematopoietic progenitors. Blood 86, 4544–4552 8541544

[RSTB20100026C112] Rodriguez-Sosa J. R. Foster R. A. Hahnel A. 2010 Development of strips of ovine testes after xenografting under the skin of mice and co-transplantation of exogenous spermatogonia with grafts. Reproduction 139, 227–235 (doi:10.1530/REP-09-0176) 19776101 10.1530/REP-09-0176

[RSTB20100026C113] Roosen-Runge E. C. Giesel L. O. Jr 1950 Quantitative studies on spermatogenesis in the albino rat. Am. J. Anat. 87, 1–30 (doi:10.1002/aja.1000870102) 14771006 10.1002/aja.1000870102

[RSTB20100026C114] Ruggiu M. Speed R. Taggart M. McKay S. J. Kilanowski F. Saunders P. Dorin J. Cooke H. J. 1997 The mouse Dazla gene encodes a cytoplasmic protein essential for gametogenesis. Nature 389, 73–77 9288969 10.1038/37987

[RSTB20100026C115] Ryu B. Y. Orwig K. E. Avarbock M. R. Brinster R. L. 2003 Stem cell and niche development in the postnatal rat testis. Dev. Biol. 263, 253–263 (doi:10.1016/j.ydbio.2003.07.010) 14597200 10.1016/j.ydbio.2003.07.010

[RSTB20100026C116] Ryu B. Y. Kubota H. Avarbock M. R. Brinster R. L. 2005 Conservation of spermatogonial stem cell self-renewal signaling between mouse and rat. Proc. Natl Acad. Sci. USA 102, 14 302–14 307 (doi:10.1073/pnas.0506970102) 10.1073/pnas.0506970102 PMC1242328 16183739

[RSTB20100026C117] Sada A. Suzuki A. Suzuki H. Saga Y. 2009 The RNA-binding protein NANOS2 is required to maintain murine spermatogonial stem cells. Science 325, 1394–1398 (doi:10.1126/science.1172645) 19745153 10.1126/science.1172645

[RSTB20100026C118] Sadate-Ngatchou P. I. Payne C. J. Dearth A. T. Braun R. E. 2008 Cre recombinase activity specific to postnatal, premeiotic male germ cells in transgenic mice. Genesis 46, 738–742 (doi:10.1002/dvg.20437) 18850594 10.1002/dvg.20437 PMC2837914

[RSTB20100026C119] Sakaki-Yumoto M. 2006 The murine homolog of SALL4, a causative gene in Okihiro syndrome, is essential for embryonic stem cell proliferation, and cooperates with Sall1 in anorectal, heart, brain and kidney development. Development 133, 3005–3013 (doi:10.1242/dev.02457) 16790473 10.1242/dev.02457

[RSTB20100026C120] Schlatt S. Ehmcke J. Jahnukainen K. 2009 Testicular stem cells for fertility preservation: preclinical studies on male germ cell transplantation and testicular grafting. Pediatr. Blood Cancer 53, 274–280 (doi:10.1002/pbc.22002) 19415740 10.1002/pbc.22002

[RSTB20100026C121] Schrans-Stassen B. H. van de Kant H. J. de Rooij D. G. van Pelt A. M. 1999 Differential expression of c-kit in mouse undifferentiated and differentiating type A spermatogonia. Endocrinology 140, 5894–5900 (doi:10.1210/en.140.12.5894) 10579355 10.1210/endo.140.12.7172

[RSTB20100026C122] Seandel M. 2007 Generation of functional multipotent adult stem cells from GPR125+ germline progenitors. Nature 449, 346–350 (doi:10.1038/nature06129) 17882221 10.1038/nature06129 PMC2935199

[RSTB20100026C123] Shinohara T. Avarbock M. R. Brinster R. L. 1999 beta1- and alpha6-Integrin are surface markers on mouse spermatogonial stem cells. Proc. Natl Acad. Sci. USA 96, 5504–5509 (doi:10.1073/pnas.96.10.5504) 10318913 10.1073/pnas.96.10.5504 PMC21889

[RSTB20100026C124] Shinohara T. Orwig K. E. Avarbock M. R. Brinster R. L. 2000 Spermatogonial stem cell enrichment by multiparameter selection of mouse testis cells. Proc. Natl Acad. Sci. USA 97, 8346–8351 (doi:10.1073/pnas.97.15.8346) 10900001 10.1073/pnas.97.15.8346 PMC26950

[RSTB20100026C125] Smith L. G. Weissman I. L. Heimfeld S. 1991 Clonal analysis of hematopoietic stem-cell differentiation *in vivo*. Proc. Natl Acad. Sci. USA 88, 2788–2792 (doi:10.1073/pnas.88.7.2788) 1672767 10.1073/pnas.88.7.2788 PMC51324

[RSTB20100026C126] Snedaker A. K. Honaramooz A. Dobrinski I. 2004 A game of cat and mouse: xenografting of testis tissue from domestic kittens results in complete cat spermatogenesis in a mouse host. J. Androl. 25, 926–930 15477365 10.1002/j.1939-4640.2004.tb03163.x

[RSTB20100026C127] Spangrude G. J. 1989 Enrichment of murine haemopoietic stem cells: diverging roads. Immunol. Today 10, 344–350 (doi:10.1016/0167-5699(89)90192-8) 2572235 10.1016/0167-5699(89)90192-8

[RSTB20100026C128] Suzuki A. Tsuda M. Saga Y. 2007 Functional redundancy among Nanos proteins and a distinct role of Nanos2 during male germ cell development. Development 134, 77–83 (doi:10.1242/dev.02697) 17138666 10.1242/dev.02697

[RSTB20100026C129] Suzuki H. Sada A. Yoshida S. Saga Y. 2009 The heterogeneity of spermatogonia is revealed by their topology and expression of marker proteins including the germ cell-specific proteins Nanos2 and Nanos3. Dev. Biol. 336, 222–231 19818747 10.1016/j.ydbio.2009.10.002

[RSTB20100026C130] Tanaka S. S. Toyooka Y. Akasu R. Katoh-Fukui Y. Nakahara Y. Suzuki R. Yokoyama M. Noce T. 2000 The mouse homolog of *Drosophila vasa* is required for the development of male germ cells. Genes Dev. 14, 841–853 10766740 PMC316497

[RSTB20100026C131] Tegelenbosch R. A. de Rooij D. G. 1993 A quantitative study of spermatogonial multiplication and stem cell renewal in the C3H/101 F1 hybrid mouse. Mutat. Res. 290, 193–200 7694110 10.1016/0027-5107(93)90159-d

[RSTB20100026C132] Tohonen V. Ritzen E. M. Nordqvist K. Wedell A. 2003 Male sex determination and prenatal differentiation of the testis. In The developing testis, vol. 5 (ed. Soder O.), pp. 1–23 Basel, Switzerland: Karger 10.1159/000069299 12629889

[RSTB20100026C133] Tokuda M. Kadokawa Y. Kurahashi H. Marunouchi T. 2007 CDH1 is a specific marker for undifferentiated spermatogonia in mouse testes. Biol. Reprod. 76, 130–141 (doi:10.1095/biolreprod.106.053181) 17035642 10.1095/biolreprod.106.053181

[RSTB20100026C134] Toyooka Y. Tsunekawa N. Takahashi Y. Matsui Y. Satoh M. Noce T. 2000 Expression and intracellular localization of mouse Vasa-homologue protein during germ cell development. Mech. Dev. 93, 139–149 (doi:10.1016/S0925-4773(00)00283-5) 10781947 10.1016/s0925-4773(00)00283-5

[RSTB20100026C135] Toyooka J. Tsunekawa N. Akasu R. Noce T. 2003 Embryonic stem cells can form germ cells *in vitro*. PNAS 100, 11 457–11 462 (doi:10.1073/pnas.1932826100) 10.1073/pnas.1932826100 PMC208779 14504407

[RSTB20100026C136] Tsuda M. Sasaoka Y. Kiso M. Abe K. Haraguchi S. Kobayashi S. Saga Y. 2003 Conserved role of nanos proteins in germ cell development. Science 301, 1239–1241 (doi:10.1126/science.1085222) 12947200 10.1126/science.1085222

[RSTB20100026C137] Tung P. S. Skinner M. K. Fritz I. B. 1984 Cooperativity between Sertoli cells and peritubular myoid cells in the formation of the basal lamina in the seminiferous tubule. Ann. N.Y. Acad. Sci. 438, 435–446 (doi:10.1111/j.1749-6632.1984.tb38304.x) 6598327 10.1111/j.1749-6632.1984.tb38304.x

[RSTB20100026C138] van Bragt M. P. Roepers-Gajadien H. L. Korver C. M. Bogerd J. Okuda A. Eggen B. J. de Rooij D. G. van Pelt A. M. 2008 Expression of the pluripotency marker UTF1 is restricted to a subpopulation of early A spermatogonia in rat testis. Reproduction 136, 33–40 (doi:10.1530/REP-07-0536) 18390688 10.1530/REP-07-0536

[RSTB20100026C139] van der Wee K. S. Johnson E. W. Dirami G. Dym T. M. Hofmann M. C. 2001 Immunomagnetic isolation and long-term culture of mouse type A spermatogonia. J. Androl. 22, 696–704 11451367

[RSTB20100026C140] Wang P. J. McCarrey J. R. Yang F. Page D. C. 2001 An abundance of X-linked genes expressed in spermatogonia. Nat. Genet. 27, 422–426 (doi:10.1038/86927) 11279525 10.1038/86927

[RSTB20100026C141] Wu Q. Chen X. Zhang J. Loh Y. H. Low T. Y. Zhang W. Sze S. K. Lim B. Ng H. H. 2006 Sall4 interacts with Nanog and co-occupies Nanog genomic sites in embryonic stem cells. J. Biol. Chem. 281, 24 090–24 094 (doi:10.1074/jbc.C600122200) 10.1074/jbc.C600122200 16840789

[RSTB20100026C142] Yamaguchi Y. L. Tanaka S. S. Kasa M. Yasuda K. Tam P. P. Matsui Y. 2006 Expression of low density lipoprotein receptor-related protein 4 (Lrp4) gene in the mouse germ cells. Gene Expr. Patterns 6, 607–612 (doi:10.1016/j.modgep.2005.11.013) 16434236 10.1016/j.modgep.2005.11.013

[RSTB20100026C143] Yang J. 2008 SALL4 is a key regulator of survival and apoptosis in human leukemic cells. Blood 112, 805–813 (doi:10.1182/blood-2007-11-126326) 18487508 10.1182/blood-2007-11-126326 PMC2481537

[RSTB20100026C144] Yeh J. R. Zhang X. Nagano M. C. 2007 Establishment of a short-term *in vitro* assay for mouse spermatogonial stem cells. Biol. Reprod. 77, 897–904 17687116 10.1095/biolreprod.107.063057

[RSTB20100026C145] Yeom Y. I. Fuhrmann G. Ovitt C. E. Brehm A. Ohbo K. Gross M. Hubner K. Scholer H. R. 1996 Germline regulatory element of Oct-4 specific for the totipotent cycle of embryonal cells. Development 122, 881–894 8631266 10.1242/dev.122.3.881

[RSTB20100026C146] Ying Y. Qi X. Zhao G. Q. 2001 Induction of primordial germ cells from murine epiblasts by synergistic action of BMP4 and BMP8B signaling pathways. Proc. Natl Acad. Sci. USA 98, 7858–7862 (doi:10.1073/pnas.151242798) 11427739 10.1073/pnas.151242798 PMC35432

[RSTB20100026C147] Yoon K. A. Chae Y. M. Cho J. Y. 2009 FGF2 stimulates SDF-1 expression through the Erm transcription factor in Sertoli cells. J. Cell Physiol. 220, 245–256 (doi:10.1002/jcp.21759) 19301256 10.1002/jcp.21759

[RSTB20100026C148] Yoshida S. Takakura A. Ohbo K. Abe K. Wakabayashi J. Yamamoto M. Suda T. Nabeshima Y. 2004 Neurogenin3 delineates the earliest stages of spermatogenesis in the mouse testis. Dev. Biol. 269, 447–458 (doi:10.1016/j.ydbio.2004.01.036) 15110712 10.1016/j.ydbio.2004.01.036

[RSTB20100026C149] Yoshida S. Sukeno M. Nakagawa T. Ohbo K. Nagamatsu G. Suda T. Nabeshima Y. 2006 The first round of mouse spermatogenesis is a distinctive program that lacks the self-renewing spermatogonia stage. Development 133, 1495–1505 (doi:10.1242/dev.02316) 16540512 10.1242/dev.02316

[RSTB20100026C150] Yoshida S. Nabeshima Y. Nakagawa T. 2007 *a* Stem cell heterogeneity: actual and potential stem cell compartments in mouse spermatogenesis. Ann. N.Y. Acad. Sci. 1120, 47–58 (doi:10.1196/annals.1411.003) 17905929 10.1196/annals.1411.003

[RSTB20100026C151] Yoshida S. Sukeno M. Nabeshima Y. 2007 *b* A vasculature-associated niche for undifferentiated spermatogonia in the mouse testis. Science 317, 1722–1726 (doi:10.1126/science.1144885) 17823316 10.1126/science.1144885

[RSTB20100026C152] Yoshimizu T. 1999 Germline-specific expression of the Oct-4/green fluorescent protein (GFP) transgene in mice. Dev. Growth Differ. 41, 675–684 (doi:10.1046/j.1440-169x.1999.00474.x) 10646797 10.1046/j.1440-169x.1999.00474.x

[RSTB20100026C153] Yoshinaga K. Nishikawa S. Ogawa M. Hayashi S. Kunisada T. Fujimoto T. 1991 Role of c-kit in mouse spermatogenesis: identification of spermatogonia as a specific site of c-kit expression and function. Development 113, 689–699 1723681 10.1242/dev.113.2.689

[RSTB20100026C154] Yuri S. 2009 Sall4 is essential for stabilization, but not for pluripotency, of embryonic stem cells by repressing aberrant trophectoderm gene expression. Stem Cells 27, 796–805 (doi:10.1002/stem.14) 19350679 10.1002/stem.14

[RSTB20100026C155] Zeng W. Avelar G. F. Rathi R. Franca L. R. Dobrinski I. 2006 The length of the spermatogenic cycle is conserved in porcine and ovine testis xenografts. J. Androl. 27, 527–533 16598031 10.2164/jandrol.05143

[RSTB20100026C156] Zhang X. Ebata K. T. Nagano M. C. 2003 Genetic analysis of the clonal origin of regenerating mouse spermatogenesis following transplantation. Biol. Reprod. 69, 1872–1878 (doi:10.1095/biolreprod.103.019273) 12904317 10.1095/biolreprod.103.019273

[RSTB20100026C157] Zhang J. 2006 Sall4 modulates embryonic stem cell pluripotency and early embryonic development by the transcriptional regulation of Pou5f1. Nat. Cell Biol. 8, 1114–1123 (doi:10.1038/ncb1481) 16980957 10.1038/ncb1481

[RSTB20100026C158] Zheng K. Wu X. Kaestner K. H. Wang P. J. 2009 The pluripotency factor LIN28 marks undifferentiated spermatogonia in mouse. BMC Dev. Biol. 9, 38 (doi:10.1186/1471-213X-9-38) 19563657 10.1186/1471-213X-9-38 PMC2719617

